# Hemorrhage and necrosis of the liver caused by hepatic arteriovenous malformations in a fetus: A case report

**DOI:** 10.1097/MD.0000000000033380

**Published:** 2023-03-24

**Authors:** Wei Bian, Jun Yuan, Yuan Yao

**Affiliations:** a Department of Radiology, Jiaxing Maternity and Child Health Care Hospital, Jiaxing, China; b Department of Pathology, Jiaxing Maternity and Child Health Care Hospital, Jiaxing, China.

**Keywords:** fetal reduction, general genetics, gynecologic imaging, hepatic, obstetrics: diagnostic ultrasound and prenatal diagnosis

## Abstract

Hepatic arteriovenous malformations (HAVMs) are a rare disorder reported in association with hereditary hemorrhagic telangiectasia (HHT), known as Rendu-Osler-Weber syndrome. HAVMs are usually detected in adulthood.

**Patient concerns:**

A 29-year-old pregnant woman underwent a routine prenatal examination at 37 weeks of pregnancy.

**Diagnosis and interventions:**

There were fetal liver anomalies detected by prenatal ultrasonography and were managed. Furthermore, a hepatic mass was detected and was subsequently analyzed by fetal magnetic resonance imaging. There were no typical imaging findings in this case which was once misdiagnosed as a hepatoblastoma.

**Outcomes:**

Considering the massive hepatic lesion, labor induction was performed on a pregnant woman to avoid adverse maternal and fetal outcomes. Histopathological examination confirmed the diagnosis of HAVMs. Lesions detected by imaging were determined to be hemorrhagic and necrotic.

**Lessons:**

Prenatal hepatic hemorrhage and necrosis due to an arteriovenous malformation are rare. The authors describe their observations and results.

## 1. Introduction

Hepatic arteriovenous malformations (HAVMs) are a rare disorder reported in association with hereditary hemorrhagic telangiectasia (HHT), known as Rendu-Osler-Weber syndrome.^[1,2]^ This paper reports the first case of hemorrhage and necrosis of the liver caused by HAVMs, confirmed by histopathological examination in a fetus with complete imaging data.

Our case was presented with predominantly hemorrhage and necrosis caused by HAVMs that were not correctly diagnosed by ultrasonography and resonance imaging (MRI) imaging. Thus, this presentation aims to improve the understanding of this disease for sonographers and radiologists to better understand the prenatal diagnosis. Timely and correct diagnosis would allow better prenatal counseling and management decisions.

## 2. Case report

**Figure 1.**
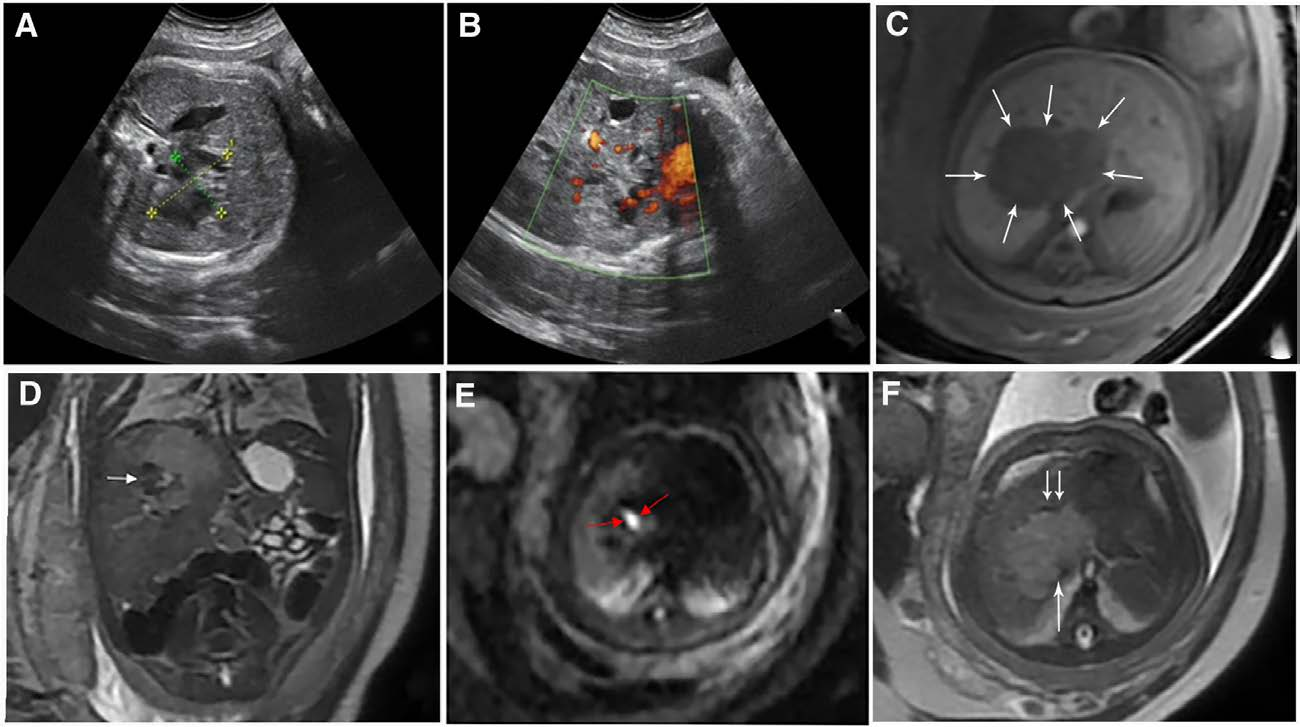
Prenatal ultrasonography and magnetic resonance imaging (MRI) findings. (A) Ultrasonography showing the presence of a continuous-space lesion measuring 4.4 cm × 3.2 cm with mixed echo in the liver. (B) CDFI detected punctuated blood flow in the mass and a short bar blood flow signal around the nodule. (C) *T*1-weighted axial MRI showing a well-defined lesion (arrow). (D) *T*2-weighted coronal MRI demonstrating hypointense in the hyperintense area (arrow). (E) Diffusion-weighted imaging revealing minimal diffusion restriction and partial diffusion restriction (red arrow). (F) *T*1-weighted axial MRI showing empty vessels around the mass (double arrows) and the inferior vena cava deformation next to the mass (long arrow).

A 29-year-old pregnant woman who was asymptomatic and attending routine antenatal visits had a routine ultrasound examination in the 37^th^ week of pregnancy. Real-time ultrasonography revealed an irregular abdominal shape and an unclear margin mass, measuring 4.4 cm × 3.2 cm × 4 cm, located in the right lobe of the fetal liver. The mass had a mixed echogenicity (hyperechoic and or anechoic pattern) (Fig. 1A). Color doppler ultrasonography revealed punctuated blood flow in the mass and a short bar blood flow signal around the nodule (Fig. 1B).

Fetal MRI examination was performed on a 1.5T MRI scanner (SINGA™ Voyager, GE Health care) for further evaluation. MRI revealed a relatively clear well-circumscribed mass with mixed signals measuring 4.6 cm × 3.3 cm × 3.5 cm segments IVa and VIII of the liver. The lesion was of heterogeneous signal intensity, *T*1 hypointense (Fig. 1C), *T*2 hyperintense mixed with patchy hypointense (Fig. 1D), and with minimal diffusion restriction partially (Fig. 1E). The axial single-shot fast spin-echo *T*2 weighted imaging (SSFSE T2WI) sequence revealed empty vascular around the mass and deformation of the inferior vena cava next to the mass (Fig. 1F). In summary, the lesion was diagnosed as hepatoblastoma.

**Figure 2.**
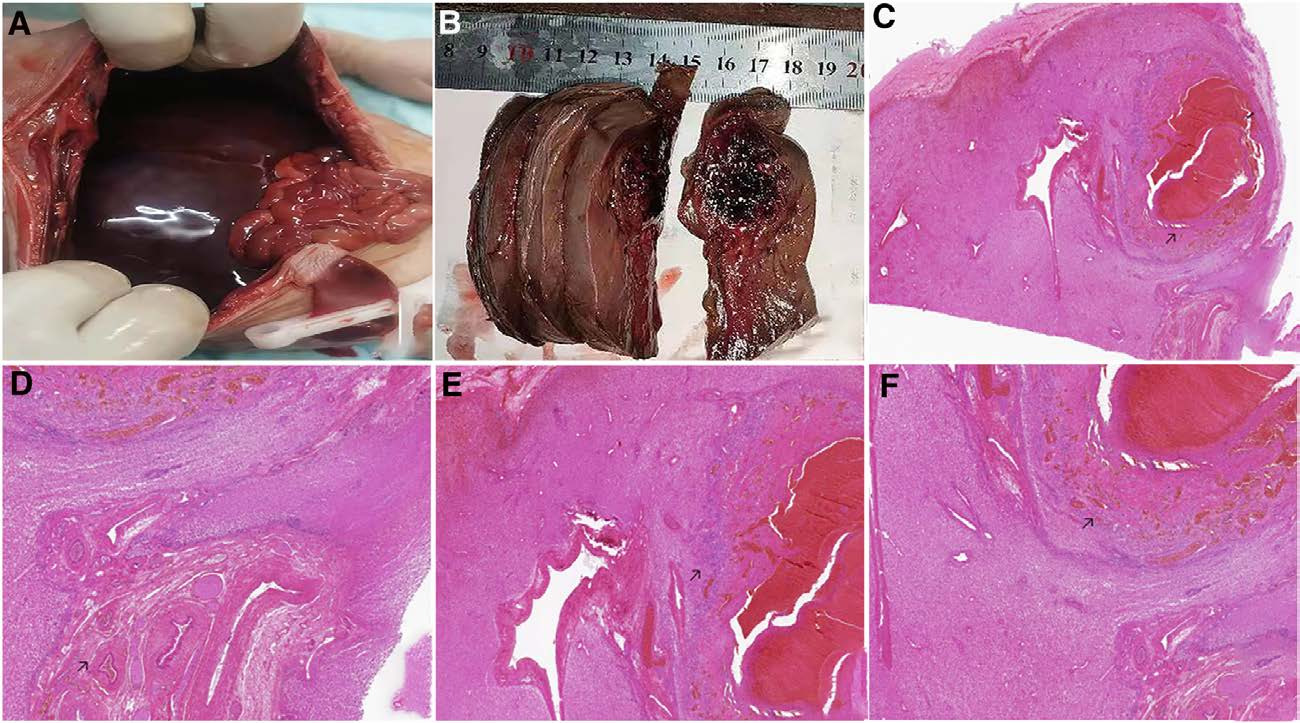
Pathological examination and hematoxylin-eosin staining (HE staining). (A) The macroscopic assessment showing hyperemia and congestion of the liver. (B) Macroscopic slices revealing hemorrhage and necrosis in the liver. (C–F) Hematoxylin and eosin staining of the liver showing hepatic vessel dilation and bleeding, hemorrhage and necrosis (magnification × 40) (C, arrow), hepatic arteriovenous malformations (magnification × 100) (D, arrow), small bile duct reactive hyperplasia (magnification × 100) (E, arrow), cavernous hemangioma-like changes (magnification × 40) (F, arrow).

**Figure 3.**
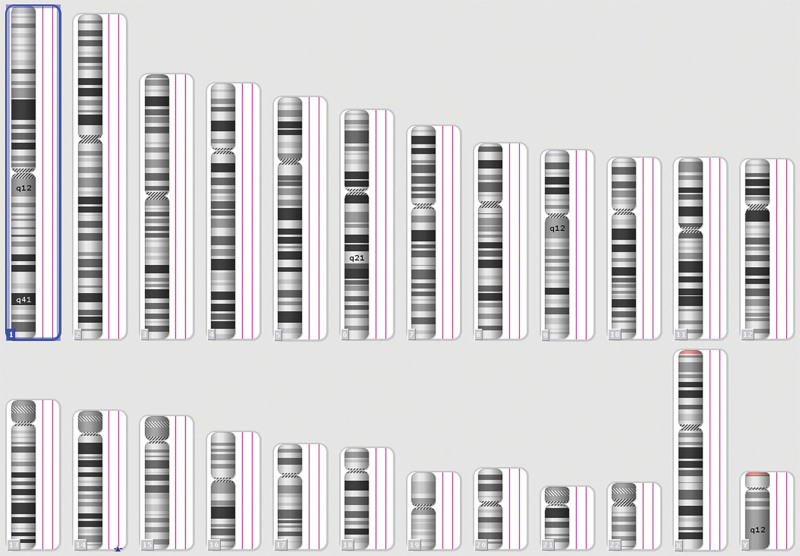
Genetic microarray analysis revealing no chromosome number copy number gain or loss.

The parents of fetus opted for pregnancy termination and agreed to an autopsy because of fear of a bad prognosis. Gross fetus samples showed hepatomegaly with a size of 12 cm × 8 cm × 4 cm (Fig. 2A) and showed hemorrhage and necrosis after dissection (Fig. 2B). Routine histopathological examination (Fig. 2C–F) displayed arteriovenous malformations in the liver and necrosis next to it. Hematoxylin and eosin staining images revealed small bile duct reactive hyperplasia around the hemorrhage and necrosis lesion, as well as cavernous hemangioma-like changes in the lesion margin. The final pathological diagnosis was hepatic arteriovenous malformation with hemorrhage and necrosis of surrounding tissues. The gene chip assay of tissue indicated no apparent abnormal findings (Fig. 3).

## 3. Discussion and conclusion

HAVMs are rare congenital lesions consisting of hepatic artery-portal vein fistula and hepatic artery-hepatic venous malformation. There have been only 8 reports of HAVMs in fetuses with typical imaging in ultrasonography in the previous literature,^[3,4]^ but no MRI imaging data were provided. HAVMs are reported to be associated with HHT, which is inherited as an autosomal dominant disease caused by mutations in ENG, ACVRL1, or SMAD-4 genes.^[5]^ HHT comprised a triad of epistaxis, telangiectasia, and suitable family history involving the mucocutaneous tissues, brain, lungs, and liver.^[2,6]^ In our case, the fetus had no family history of inherited diseases through medical record review. Although the gene chip assay was normal in our case, whole-exome sequencing should be recommended because of insufficient deception. Mucosal bleeding points and vascular malformations of other organs had not been detected.

In HAVMs, abnormal blood circulation in the liver causes hepatomegaly and portal hypertension.^[7]^ In our study, the fetus presented with significantly larger hepatomegaly than observed at the same gestational age. In adults, like other arteriovenous malformations, HAVMs show that arteries and veins communicate directly with high-pressure arteries feeding into low-pressure veins via malformed vascular structures without a capillary.^[8]^ However, the blood supply of fetal liver, different from that of the adult, is dominantly supplied by the umbilical vein with a higher pressure than hepatic artery and vein.^[6]^ Doppler ultrasonography plays a key role in detecting hemodynamic changes. Enhanced CT or MRI examinations are important for detecting arteriovenous malformations, including feeding arteries, intra-tumoral vasculature, and draining veins. Because of radiation protection and disabling of contrast agents for the fetus, indirect signs such as hemorrhage, necrosis mass, and disordered vessels around the lesion were detected instead of typical ultrasonography and MRI imaging signs. The imaging findings of this case are similar to the cerebral arteriovenous malformations that were finally diagnosed using DSA reported by Eguchi et al^[9]^ High-pressure blood flow directly into vascular structures can disrupt the thin vessel with defects in smooth muscle and can ultimately cause hemorrhage.^[10]^ The steal phenomenon caused by arteriovenous malformations leads to insufficient blood supply to other liver parts and causes necrosis.^[11]^ In our case, ultrasound hypoechoic areas, T1WI hypointensity, and T2WI hyperintensity suggest old hemorrhagic necrosis, whereas ultrasound hyperechoic areas and T2WI patchy hypointensity suggest hemorrhage or hemosiderin deposition, consistent with gross specimens. The short bar blood flow signal around the nodule in color doppler ultrasonography and empty vascular around the mass on T2WI may suggest the presence of vascular malformations.

Fetal liver lesions are rare, and differential diagnoses of hepatic arteriovenous malformations mainly include hepatic hemangioma, hepatoblastoma, and metastatic malignant tumors. Similar to our case, hepatoblastoma was predominantly *T*2-hyperintense and *T*1-hypointense with heterogeneity areas due to necrosis, hemorrhage, or calcification in MRI imaging.^[12]^ However, hepatoblastoma was typically a hyperechoic solid mass with aggressive features such as rapid growth or metastases, which are helpful for differential diagnosis.^[12,13]^ Diffusion restriction (high in diffusion-weighted imaging) may also contribute to misdiagnosis. Malignant tumors like hepatoblastoma have highly cellular tissue and several cell membranes per unit volume, limiting water molecules diffusion into the extracellular space.^[14]^ However, the diffusion restriction may indicate edematous areas of lesion or hemorrhage in the acute or subacute phase.^[15]^ The deformation of inferior vena cava in *T*2-weighted imaging that was misidentified as an invasion of hepatoblastoma tumor was caused by compression of hemorrhage and necrosis. In addition to hemorrhage and necrosis, HAVMs can result in high-output heart failure.^[16]^ In this case, cardiac abnormalities have not yet been described by ultrasonography. Additionally, multiple or large lesions of hepatic arteriovenous malformations can lead to significant complications, including hepatic encephalopathy, biliary ischemia, liver failure, and so on.^[17]^

HAVM treatment mainly includes interventional treatment and surgical resection. Several molecular targeted drugs, such as the anti-VEGF inhibitor bevacizumab, are reported to improve the increased cardiac output due to HAVMs.^[18,19]^ The best way is liver transplantation,^[20]^ which increases the family’s financial burden. preoperative diagnosis in our case seems to be very difficult because of the limitations of fetal ultrasonography and magnetic resonance imaging. The confirmed diagnosis of hepatic arteriovenous malformations in a fetus should rely on the common decision of imaging examinations, gross sample observation, and pathological examinations. Besides typical signs in imaging, sonographers and radiologists should improve the understanding of HAVMs and consider the possibility of tissue hemorrhage and necrosis caused by HAVMs when discovering liver disease during prenatal examinations.

preoperative diagnosis in our case seems to be very difficult because of the limitations of fetal ultrasonography and magnetic resonance imaging. The confirmed diagnosis of hepatic arteriovenous malformations in a fetus should rely on the common decision of imaging examinations, gross sample observation, and pathological examinations. Besides typical signs in imaging, sonographers and radiologists should improve the understanding of HAVMs and consider the possibility of tissue hemorrhage and necrosis caused by HAVMs when discovering liver disease during prenatal examinations.

It is common to misdiagnose HAVMs due to their nonspecific presentation. This case highlights the importance of having a high index of suspicion when diagnosing HAVMs.

## Author contributions

**Conceptualization:** Wei Bian

**Data curation:** Wei Bian, Jun Yuan.

**Writing – original draft:** Wei Bian.

**Writing – review & editing:** Yuan Yao.

## References

[1] Sukkarieh F Brasseur P. [Pulmonary and hepatic arteriovenous malformations in a case of Rendu-Osler disease]. J Radiol. 2003;84:405–8. 12759655

[2] Shovlin CL Guttmacher AE Buscarini E. Diagnostic criteria for hereditary hemorrhagic telangiectasia (Rendu-Osler-Weber syndrome). Am J Med Genet. 2000;91:66–7. 10751092 10.1002/(sici)1096-8628(20000306)91:1<66::aid-ajmg12>3.0.co;2-p

[3] Wu Y Zhou L Chen L. Correlations among congenital hepatic shunt, absent ductus venosus, and umbilical vein shunt revealed by prenatal ultrasound. Fetal Diagn Ther. 2020;47:237–44. 31553987 10.1159/000502182

[4] Demirci O Celayir A. Prenatal diagnosis and treatment of intrahepatic arteriovenous fistulas: case reports and the literature review. J Matern Fetal Neonatal Med. 2022;35:837–45. 32241194 10.1080/14767058.2020.1731466

[5] Bofarid S Hosman AE Mager JJ. Pulmonary vascular complications in hereditary hemorrhagic telangiectasia and the underlying pathophysiology. Int J Mol Sci. 2021;22:3471. 33801690 10.3390/ijms22073471 PMC8038106

[6] Albers BK Khanna G. Vascular anomalies of the pediatric liver. Radiographics. 2019;39:842–56. 31059404 10.1148/rg.2019180146

[7] Tiwari C Nagdeve N Saoji R. Congenital hepatic arteriovenous malformation presenting as isolated massive hepatomegaly in an otherwise healthy neonate: a case report. J Mother Child. 2020;24:67–70. 33074180 10.34763/jmotherandchild.2020241.2002.000008 PMC8518103

[8] Ryan SD Nambiar A Maingard J. Endovascular embolization of canine hepatic arteriovenous malformations using precipitating hydrophobic injectable liquid (PHIL) liquid embolic agent: a proof-of-concept study. CVIR Endovasc. 2019;2:27. 32026126 10.1186/s42155-019-0070-4 PMC6966389

[9] Eguchi S Aihara Y Yamaguchi K. Limitations of fetal ultrasonography and magnetic resonance imaging in prenatal diagnosis of congenital cerebral arteriovenous malformations with hemorrhagic onset. J Neurosurg Pediatr. 2012;10:154–8. 22725727 10.3171/2012.4.PEDS11517

[10] Zyck S Sampath R. Arteriovenous Malformations. Treasure Island (FL): StatPearls Publishing; 2022. 30285374

[11] Sanchez-Morales GE Anaya-Ayala JE Serrano-Cueva MA. Hand ischemia due to steal syndrome associated with multiple arteriovenous malformations in a patient with Parkes-Weber syndrome. J Hand Surg Asian Pac Vol. 2019;24:89–92. 30760156 10.1142/S2424835519720019

[12] Birkemeier KL. Imaging of solid congenital abdominal masses: a review of the literature and practical approach to image interpretation. Pediatr Radiol. 2020;50:1907–20. 33252758 10.1007/s00247-020-04678-1

[13] Cass DL. Fetal abdominal tumors and cysts. Transl Pediatr. 2021;10:1530–41. 34189111 10.21037/tp-20-440 PMC8192983

[14] Gawande RS Gonzalez G Messing S. Role of diffusion-weighted imaging in differentiating benign and malignant pediatric abdominal tumors. Pediatr Radiol. 2013;43:836–45. 23666206 10.1007/s00247-013-2626-0

[15] Renard D Tatu L Collombier L. Cerebral amyloid angiopathy and cerebral amyloid angiopathy-related inflammation: comparison of hemorrhagic and DWI MRI features. J Alzheimers Dis. 2018;64:1113–21. 30010128 10.3233/JAD-180269

[16] Cusumano LR Tesoriero JA Wilsen CB. Predictors of heart failure symptoms in hereditary hemorrhagic telangiectasia patients with hepatic arteriovenous malformations. Orphanet J Rare Dis. 2021;16:478. 34794458 10.1186/s13023-021-02109-4 PMC8600745

[17] Nunes DSI Matos C Correia F. Osler-Weber-Rendu syndrome with severe hepatic manifestations: a rare clinical case. Eur J Case Rep Intern Med. 2020;7:1831. 10.12890/2020_001831 PMC7655005 33194857

[18] Olsen LB Kjeldsen AD Poulsen MK. High output cardiac failure in 3 patients with hereditary hemorrhagic telangiectasia and hepatic vascular malformations, evaluation of treatment. Orphanet J Rare Dis. 2020;15:334. 33243256 10.1186/s13023-020-01583-6 PMC7691053

[19] Snodgrass RO Chico T Arthur HM. Hereditary haemorrhagic telangiectasia, an inherited vascular disorder in need of improved evidence-based pharmaceutical interventions. Genes (Basel). 2021;12:2. 10.3390/genes12020174 PMC7911152 33513792

[20] Lee M Sze DY Bonham CA. Hepatic arteriovenous malformations from hereditary hemorrhagic telangiectasia: treatment with liver transplantation. Dig Dis Sci. 2010;55:3059–62. 20844961 10.1007/s10620-010-1353-8

